# Is *Candida albicans* an opportunistic oncogenic pathogen?

**DOI:** 10.1371/journal.ppat.1010413

**Published:** 2022-04-14

**Authors:** Ahmed S. Sultan, Vasileios Ionas Theofilou, Areej Alfaifi, Daniel Montelongo-Jauregui, Mary-Ann Jabra-Rizk

**Affiliations:** 1 Department of Oncology and Diagnostic Sciences, School of Dentistry, University of Maryland, Baltimore, Maryland, United States of America; 2 University of Maryland Greenebaum Cancer Center, Baltimore, Maryland, United States of America; 3 Department of Restorative and Prosthetic Dental Sciences, College of Dentistry King Saud bin Abdulaziz University for Health Sciences, Riyadh, Saudi Arabia; 4 Department of Microbiology and Immunology, School of Medicine, University of Maryland, Baltimore, Maryland, United States of America; Vallabhbhai Patel Chest Institute, INDIA

## Introduction

Until recent years, *Candida albicans* had fundamentally been linked to cancer as an opportunistic pathogen that takes advantage of an immunosuppressed state [1]. However, there is a growing body of evidence that this dimorphic fungal species may be capable of promoting cancer [2]. *C*. *albicans* is a normal commensal of the human body and therefore does not induce damage. However, as an opportunistic pathogen, *C*. *albicans* is capable of becoming pathogenic when the host defenses are weakened, causing an array of infections ranging from mucosal to systemic [1]. Oral candidiasis, commonly known as “thrush,” is one of the most common infections of the oral cavity characterized by fungal overgrowth and infiltration of superficial tissues involving the tongue and other oral mucosal sites. Among the spectrum of oral mucosal lesions associated with *Candida*, chronic hyperplastic candidiasis, also known as candidal leukoplakia, has been associated with the risk of malignant transformation to oral cancer [1,3]. The association between *Candida* and oral cancer has traditionally been a subject of debate, and many mechanisms of potential interactions between this fungal pathogen and oral carcinogenesis have been described [4]. Mounting evidence has supported a correlation between *Candida* infection and development of oral epithelial dysplasia [5], a spectrum of histopathological changes that affect the epithelial lining of the oral mucosae displaying increased risk of progression to oral squamous cell carcinoma (OSCC) or oral cancer [3]. In this article, we review prior research directly or indirectly linking *Candida* and oral cancer (**Table 1**) and posit that candidiasis may not just be randomly coexisting with oral cancer, but the pathogenetic relationship is also a dominant scenario, including the possibility that *C*. *albicans* may initiate or facilitate the development of oral cancer. Further, we describe the main proposed mechanisms by which this yeast species may induce cancer and highlight the need for further future mechanistic studies in oral carcinogenesis models to establish *C*. *albicans* as an opportunistic oncogenic pathogen.

**Table 1:** Overview of select descriptive and mechanistic evidence that directly or indirectly highlight the potential roles of *Candida* in oral carcinogenesis.

Year	First author(s)	Type of study	Key experimental approaches	Main findings
1987	Krogh [13]	Descriptive	Liquid and gas chromatography in yeast isolated from oral premalignancy patients and healthy participants	Elevated *Candida albicans* strains with nitrosation potential in oral premalignancy
1992	O’Grady [21]	Mechanistic	4NQO rat model with *Candida* coinfection	*Candida* promotes 4NQO-induced oral carcinogenesis
2002	McCullough [5]	Descriptive	Oral swish and culture of *Candida* in oral premalignancy and cancer as well as controls	Increased frequency of oral yeast carriage and colony-forming units in patients with oral epithelial dysplasia and cancer compared with controls
2009	Dwivedi [22]	Mechanistic	4NQO mouse model with *Candida* coinfection	Validation of oral cancer promoting roles of Candida in 4NQO mouse model
2013	Hebbar [6]	Descriptive	Histology (PAS stain) and oral swish with culture from oral potentially malignant disorders and oral cancer	Presence of *Candida* hyphae correlates with severity of dysplastic epithelial changes
2015	Alnuaimi [9]	Descriptive	Isolation of oral yeast and genetic identification with RT-PCR from OSCC versus control patients	*C*. *albicans* is an independent risk factor for oral cancer development Combination with alcohol generates a higher risk *C*. *albicans* genotype A predominates in oral cancer
2016	Alnuaimi [19]	Descriptive	Crystal violet staining/XTT salt reduction assays, agar plate enzyme detection method, and gas chromatography in *Candida* isolated from OSCC patients and healthy controls	Increased biofilm mass, metabolic activity, high phospholipase, and acetaldehyde production by *Candida* in oral cancer
2019	Roy [8]	Descriptive	CHROMagar assay in *Candida* isolated from oral premalignancy and cancer patients as well as healthy controls	Dysbiosis of mycobiome with emergence of *Candida krusei*, *Candida glabrata*, and *Candida tropicalis*, increasing in patients with dysplastic lesions or OSCC
2019	Ho [35]	Mechanistic	Diverse comprehensive approaches in cell cultures, murine, and zebrafish models	Virulence factor “candidalysin” activates molecular pathways that have been implicated in carcinogenesis (MAPK pathway and activation of immune responses) in an EGFR-related manner
2021	Break and Oikonomou [31]	Mechanistic	Comprehensive experimental approaches primarily in mouse models (including Aire-deficient mice), as well as cell cultures, and APECED patients	Hyperactivation of type 1 immune responses leading to epithelial destruction and subsequent *Candida* superinfection is seen in APECED syndrome, a disease which correlates with uncommon OSCC development
2022	Vadovic [23]	Mechanistic	Multiple techniques in cell lines, OSCC xenograft mouse model, and 4NQO mouse model	*Candida* induces increased migration, expression of matrix metalloproteinases, activation of epithelial to mesenchymal transition, and expression of genes implicated in metastatic processes by OSCC cells. The tumor promoting roles of *Candida* in a 4NQO model were also highlighted

4NQO, 4-nitroquinoline-1-oxide; APECED, autoimmune polyendocrinopathy–candidiasis–ectodermal dystrophy; EGFR, epidermal growth factor receptor; OSCC, oral squamous cell carcinoma; RT-PCR, real-time PCR.

### *Candida* colonization is correlated with oral premalignancy

The earliest studies investigating a potential role for oral yeast in oral carcinogenesis were mostly descriptive, relying on assessing the relative frequencies of fungal species in oral premalignancy and cancer. However, although correlative, these studies have undoubtedly demonstrated increased *Candida* colonization, as the epithelial lining of the oral mucosa alters from normal to dysplastic epithelium [5,6]. Moreover, *Candida* recovery from the oral cavity of patients with oral epithelial dysplasia [5] and the presence of hyphae in tissue sections [6] correlated with the severity of dysplastic changes. Further, sequencing techniques revealed alterations in the relative frequencies of the constituents of the oral mycobiome (fungal biome) as a whole [7]. Interestingly, differences in diversity with an abundance of less common non-*albicans* species have been observed in patients with dysplastic lesions or OSCC [8]. In the context of established oral cancer, *C*. *albicans* is considered a risk factor for oral carcinogenesis, while the combination of candidiasis with alcohol drinking generated the highest risk [9]. However, despite the copiousness of incidental evidence, the potential contribution of fungi to oral carcinogenesis remains a debatable subject.

### Initiator or facilitator?

**Fig 1:**
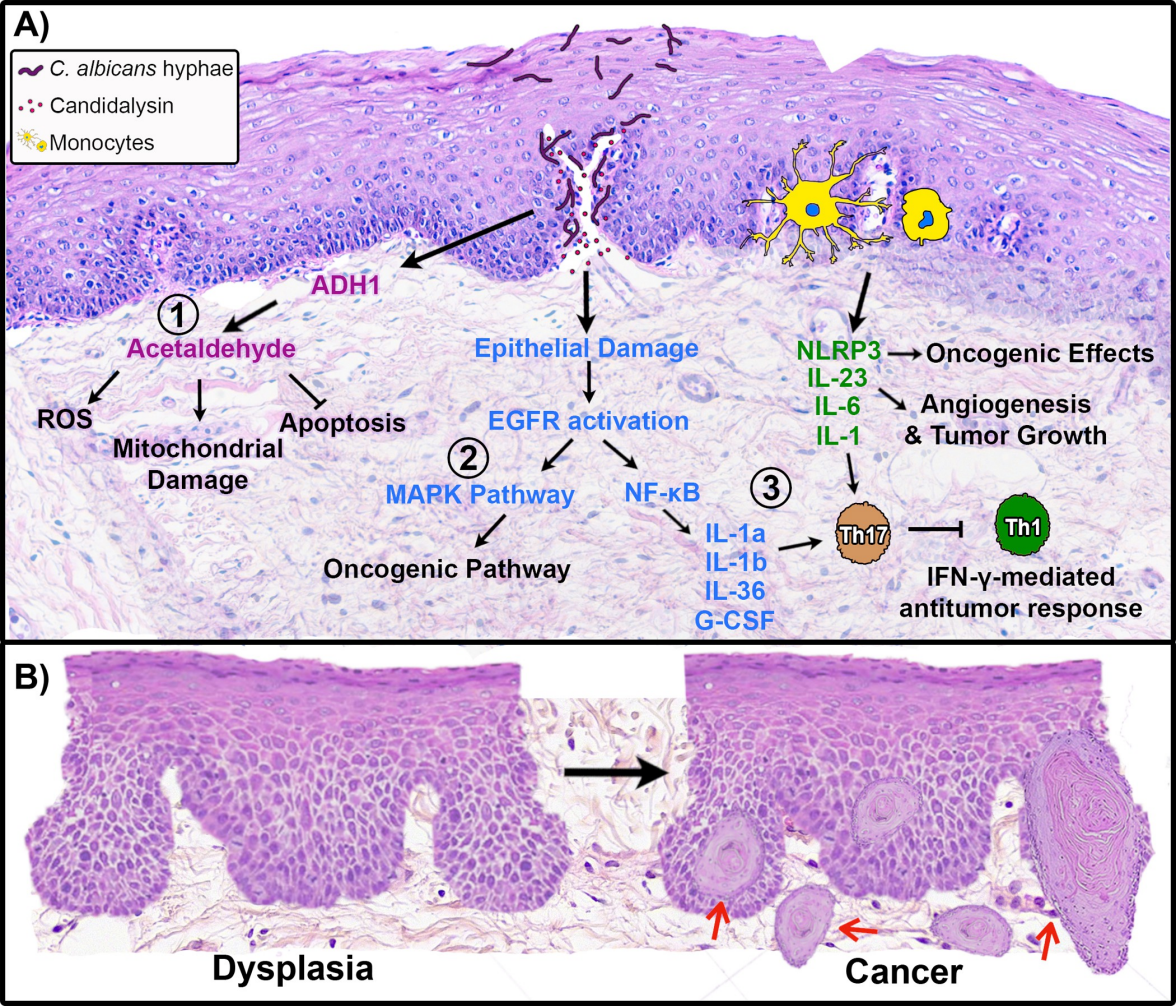
Mechanisms by which *Candida albicans* may play a role in oral cancer development. **(A) (1) Production of carcinogenic substances.** *C*. *albicans*, using the enzyme ADH1, is capable of metabolizing alcohol to acetaldehyde, which is carcinogenic. Acetaldehyde binds to proteins and DNA modifying their structure and functionality, resulting in mitochondrial damage, and also reducing antioxidant activity of glutathione leading to increased intracellular levels of ROS. These alterations may produce genome instability linked with inhibition of the apoptotic machinery ultimately leading to tumor development. **(2) Activation of oncogenic pathways in epithelial cells by candidalysin.** *C*. *albicans* secrete candidalysin toxin that damages the epithelial barrier and activates EGFR with downstream up-regulation of the MAPK pathway that has been implicated in various types of cancer. **(3) Induction of tumor-promoting immunity.** EGFR activation also causes downstream up-regulation of the NFκΒ pathway in epithelial cells resulting in the expression of IL-1a, IL-1b, IL-36, and G-CSF. Myeloid cells including antigen presenting cells and macrophages recognize *Candida* and secrete tumor promoting cytokines including IL-23, IL-6, and IL-1. Additionally, the NLRP3 inflammasome pathway is activated. Collectively, cytokines secreted by epithelial and myeloid cells result in activation of Th17 (IL-17 secreting) cells. Type 17 immune responses further support cancer progression by antagonizing Th1 (IFNγ secreting) cells. **(B)** At the oral mucosa, these tumor promoting mechanisms may have the potential of causing cytologic and architectural alterations in the oral epithelium (dysplasia), and their accumulation may lead to the development of OSCC, which is characterized by tumor islands (red arrows) invading the underlying connective tissue. ADH1, alcohol dehydrogenase 1; EGFR, epidermal growth factor receptor; G-CSF, granulocyte colony-stimulating factor; IFNγ, interferon gamma; IL, interleukin; NFκΒ, nuclear factor kappa B; OSCC, oral squamous cell carcinoma; ROS, reactive oxygen species; Th, T helper.

Although mechanisms by which bacteria and viruses stimulate cancer development are well investigated, very few studies have explored the role of fungi in this context. Neoplastic processes affecting other organs have been investigated [10], including pancreatic cancer induced by *Malassezia* genus [11]. However, conversely, the same fungus was shown to correlate with favorable prognosis [12] in oral cancer, highlighting the complex and organ-specific oncogenicity of mycobiome dysbiosis. Within the framework of oral cancer, *C*. *albicans* has primarily been studied in the context of being an “initiator” of carcinogenesis. Many possible mechanisms of diverse etiology have been implicated and are summarized in **Fig 1**. The most widely accepted hypothesis regarding the carcinogenic effect of *Candida* on the mucosal epithelium is related to the production of carcinogens such as nitrosamine [13] and acetaldehyde (a mutagenic compound that is indisputably carcinogenic) [2,14,15]. Acetaldehyde is the first metabolite of ethanol catabolism in epithelial cells and *C*. *albicans*. In the oral cavity, acetaldehyde produces DNA and protein adducts that interfere with normal DNA replication causing point mutations and chromosomal aberrations [16]. Further, acetaldehyde also affects enzymes involved in DNA repair and binds to the essential antioxidant glutathione, indirectly increasing the presence of reactive oxygen species (ROS), which are related to an increase in DNA damage. Mitochondrial damage is also induced by acetaldehyde, increasing ROS production [17,18]. In fact, *Candida* was shown to display increased metabolic activity and acetaldehyde production in oral cancer compared to healthy controls, reinforcing its potential carcinogenic role [19]. The possible oncogenic effects of *Candida* strains have been considered to be significantly affected by polymicrobial interactions, and other constituents of the microbiome seem to act antagonistically or synergistically during *Candida*-related oral carcinogenesis [20].

On the other hand, some studies described *C*. *albicans* as a promoter or “facilitator” of cancer development, rather than initiators; in one study, oral inoculation with *C*. *albicans* or administration of the carcinogen 4NQO [21] failed to cause dysplastic changes in animal models; however, in combination, oral epithelial dysplasia occured, indicating that *C*. *albicans* may have promoted dysplastic changes [21–23]. The potential tumor promoting roles of *Candida* were also confirmed by in vitro studies in oral cancer cells in which *C*. *albicans* increased the migration ability, expression of matrix metalloproteinases, secretion of oncometabolites, and expression of metastasis-related genes [23]. An underestimated alternative hypothesis is that oral candidiasis and a dysplastic epithelium are unrelated pathophysiologically or that they display the inverse cause-and-effect relationships, as *Candida* infections and premalignancy display common predisposing factors, most notably immunosuppression [24,25]. It is also important to consider that defective epithelium with destructed architecture (in the context of oral premalignancy or cancer) may enhance susceptibility to infections [26]. Nevertheless, despite the increasing evidence linking *Candida* with oral cancer, the largely descriptive nature of prior studies cannot reliably ascertain the underlying pathogenetic mechanisms that implicate *Candida* or the oral mycobiome in oral carcinogenesis.

### *Candida* and immune dysfunction during oral carcinogenesis

The transition from normal oral epithelium to dysplasia and ultimately to OSCC (Fig 1B) is a multistep process and is multifactorial in its etiopathogenesis. Owing to this multifactorial process, studies have explored the interplay between tissue inflammation, immunity, and the tumor microenvironment on etiopathogenesis [27]. In the case of *Candida* the “initiator” scenario, infection of mucosal tissue generates epithelial barrier destruction activating type 17 immune responses [28]. T helper 17 cells, a subset of CD4 T-cells, produce interleukin (IL)-17, which is required for resistance against *C*. *albicans*; therefore, Th17 immunity is the dominant response against oral candidiasis. However, other cytokines of the Th17 family, such as IL-23, promote angiogenesis and tumor growth [29]. Moreover, type 17 responses antagonize IL-12 and interferon gamma (IFNγ), both of which are crucial in Th1-type antitumor immune responses [29]. In addition to its direct effect, IL-17 can also favor cancer processes indirectly by recruiting neutrophils. Although these leukocytes are the main effector cells against *C*. *albicans*, their presence in tumor tissues also correlates with poor prognosis in some types of cancer [2].

Alternatively, an established epithelial malignancy displaying reduced levels of T-cell inflammation [30] could also possess inadequate Th17 responses, resulting in susceptibility to candidiasis. Another possibility of secondary infection of OSCC as a result of deregulated immune responses is also supported by recent evidence indicating that hyperactivation of IFNγ-induced immunity may cause epithelial destruction with subsequent *Candida* infection during autoimmune polyendocrinopathy–candidiasis–ectodermal dystrophy syndrome, a disease that also correlates with oral cancer development [31]. The potential of autoimmune hyperactivation of T cells to drive subsequent increase of fungal burden at a malignant mucosal barrier has been previously shown in oncogenesis of the esophagus; however, interestingly in this context, the recruited fungal organisms were shown to futher promote esophageal cancer development [32]. Therefore, the question remains: Does *Candida* induce epithelial damage facilitating a tumor promoting microenvironment, or does a cancerized field with immune dysfunction drive secondary susceptibility to fungal infections? And regardless, what is the role of host immune responses during these interactions? Perhaps our best understanding of the complex *Candida*–epithelial barrier–host immunity axis came about with the discovery of candidalysin.

### The newest toxin in town: Candidalysin and its oncogenic potential

A cytolytic peptide secreted by *C*. *albicans* hyphae capable of disrupting mucosal integrity [33] was recently added to the already impressive list of virulence factors in *C*. *albicans’* armamentarium. Candidalysin, a product of the expression of the *ECE1* gene [34], is considered a toxin and was shown to induce epithelial damage, activate the MAPK pathway, and induce secretion of inflammatory cytokines by epithelial cells [33], a process dependent on epidermal growth factor receptor (EGFR) signaling [35]. Additionally, candidalysin was also associated with downstream activation of type 17 immune responses [36], as well as the promotion of the NLRP3 inflammasome [37]. Given that EGFR aberrant expression and activation of the MAPK pathway [38] as well as NLRP3 [39] have been correlated with various epithelial and nonepithelial malignancies, this virulence factor was also associated with tumor-promoting immunity [40,41]. However, despite the significant indications that candidalysin may play a role in initiating or promoting oral carcinogenesis, there is no evidence that *Candida* strains that overexpress *ECE1* predominate during oral cancer. Importantly, these hypotheses have not been confirmed by mechanistic studies in animal models.

## Conclusions and future directions

To date, the exact role that the mycobiome and *Candida* in particular play in the pathogenesis of oral cancer has been a subject of disagreement. Although numerous studies have provided supporting evidence that *Candida* may initiate or promote oral epithelial oncogenesis, it is as likely that the increase in *Candida* colonization in precancerous dysplastic lesions is coincidental, as a result of an altered mucosal barrier that favors the proliferation of these common commensals. Therefore, there is a clear need for comprehensive experimental studies to confidently expound the role of *Candida* and other fungal species in oral carcinogenesis and provide mechanistic insights to deepen our understanding of the the pathogenesis of oral premalignancy and cancer with regard to its correlation to fungal dysbiosis. Regarding the tumor initiating or promoting effects of *Candida*, the possible implication of candidalysin in oral tumorigenesis should be validated by in vitro and in vivo approaches. On the other hand, the opposite scenario of passive colonization of oral premalignancy and OSCC by *Candida* has been minimally studied. Original research investigating whether epithelial changes during tumorigenesis (including altered surface receptor profile or defective intercellular communications) that may create a microenvironment that may facilitate *Candida* superinfection would also be beneficial to study. Importantly, consideration should be given to the possibility of new individualized therapeutic approaches including antifungal drugs concurrently with antitumor therapies, to minimize the risk of *C*. *albicans* and its effect in generating a protumor microenvironment.
